# Comparison of different speech tasks among adults who stutter and adults who do not stutter

**DOI:** 10.6061/clinics/2016(03)06

**Published:** 2016-03

**Authors:** Ana Paula Ritto, Julia Biancalana Costa, Fabiola Staróbole Juste, Claudia Regina Furquim de Andrade

**Affiliations:** Faculdade de Medicina da Universidade de São Paulo (FMUSP), Departamento de Fisioterapia, Fonoaudiologia e Terapia Ocupacional, São Paulo/SP, Brazil

**Keywords:** Speech, Stuttering, Models, Neurological, Feedback, Sensory

## Abstract

**OBJECTIVES:**

In this study, we compared the performance of both fluent speakers and people who stutter in three different speaking situations: monologue speech, oral reading and choral reading. This study follows the assumption that the neuromotor control of speech can be influenced by external auditory stimuli in both speakers who stutter and speakers who do not stutter.

**METHOD:**

Seventeen adults who stutter and seventeen adults who do not stutter were assessed in three speaking tasks: monologue, oral reading (solo reading aloud) and choral reading (reading in unison with the evaluator). Speech fluency and rate were measured for each task.

**RESULTS:**

The participants who stuttered had a lower frequency of stuttering during choral reading than during monologue and oral reading.

**CONCLUSIONS:**

According to the dual premotor system model, choral speech enhanced fluency by providing external cues for the timing of each syllable compensating for deficient internal cues.

## INTRODUCTION

Persistent developmental stuttering is a communication pathology characterized by involuntary speech disruptions, such as part-word (i.e., sound or syllable) repetitions, sound prolongations and blocks (postural fixations) 1. Despite the extensive number of theories relating stuttering to speech motor programming deficits 1,2,3,4,5,6,7,8, the actual mechanisms behind the speech disruptions are not yet clearly defined. Furthermore, converging studies have gathered evidence leading to the conclusion that stuttering is also somehow related to the auditory system, as verified by the reduction or elimination of observable symptoms of stuttering during different types of altered auditory feedback 9,10,11,12,13.

One of the models used to explain the stuttering mechanism is based on the hypothesis of the dual premotor system 14,15,16,17. This hypothesis defines two parallel premotor circuits: the medial system (the basal ganglia and supplementary motor area) and the lateral system (the cerebellum and lateral premotor cortex). The medial system is responsible for controlling speech initiation signals, i.e., the neural activation of motor planning for speech. The lateral system is responsible for motor activation in response to external sensory stimuli.

The supplementary motor area (SMA) is an area located on the superior frontal gyrus and constitutes the medial part of Brodmann's area. The literature suggests that the SMA is involved in self-initiated, complex and sequential movements 18. The basal ganglia are a set of interconnected subcortical structures involved in an extensive number of functions, from cognitive and motivational functions to motor control, including the automation of fast motor sequences 19. These functions suggest an important role for the basal ganglia and SMA in speech. Speech is a motor sequence that requires accurate synchronization. Accordingly, fluent speech requires accurate signals for speech initiation and temporality. The model suggests that the SMA is responsible for the motor programming of each speech segment and the basal ganglia help this process by providing internal timing cues to facilitate the initiation of speech movements. Hence, stuttering would be caused by a disturbance in the medial system, especially in the basal ganglia 16,17,20.

In line with this notion, the medial system would be active during spontaneous speech. However, during some speech modes, such as speech that is synchronized with external stimuli, the external timing cues can compensate for deficient internal cues from the basal ganglia to the SMA. In these cases, the model suggests that the dominance for speech timing is shifted from the medial to the lateral system. According to the model, the lateral system can also be responsible for the temporal control of speech rate in the absence of external sensory stimuli, but only in situations that require more attention to some particular aspect of speech, for example, when the person is using a speech pattern different from the usual (such as a different accent) or speaking at an exaggeratedly pace or during dramatization 16,17.

The present study tested the assumption that the neuromotor control of speech can be influenced by external auditory stimuli in both speakers who stutter and speakers who do not stutter. In this study, we compared the performance of fluent speakers and people who stutter in three different speaking situations: monologue speech, oral reading and choral reading. During choral reading, an external sensory stimuli was offered. We hypothesized that choral reading would improve fluency for all speakers and differ from monologue and oral reading, which should produce similar results for speech fluency according to the model.

## METHOD

### Participants

Participants for this study were 34 normal-hearing adults who were native speakers of Brazilian Portuguese and assigned to two groups. The research group, Group 1, was composed of 17 adults who stutter (14 males, 3 females; age range: 20 to 50 years, mean age 33.05 years, standard deviation 9.49). The participants were recruited from the Department of Physiotherapy, Speech-Language and Hearing Sciences and Occupational Therapy, School of Medicine, University of São Paulo (São Paulo, Brazil). Group 1 participants scored 25 points or more (diagnosed with at least “moderate” stuttering) on the Stuttering Severity Instrument – 3 , SSI-3 (Pro-Ed: Austin, Texas, United States of America) 21, did not have a history of other communication disorders and were not diagnosed with neurological and/or degenerative diseases. The control group, Group 2, comprised 17 fluent adults paired with the adults in Group 1 by age and gender. Group 2 participants scored less than 10 points on the SSI-3 21 (normal speech fluency) and did not have a history of any communication disorders or neurological and/or degenerative diseases. All participants had normal or corrected-to-normal vision and had received at least a high school diploma.

This study received prior approval by the School of Medicine at University of São Paulo Ethics Committee (CEP – FMUSP 275/14), and informed consent was obtained from all participants.

### Data collection and analysis

Three speech samples from each participant were videotaped using a Sony® DRC-SR62 video camera: monologue speech, oral reading and choral reading. For the monologue task, participants were given a picture as a stimulus and were instructed to freely discuss it. For both the oral reading and choral reading tasks, the participants read aloud different 200-syllable texts with similar themes and syntactic complexity. During the oral reading, the reading was performed solo (i.e., the participant was the only one reading); for the choral reading, the participant and the evaluator read the text simultaneously. For the choral reading condition, the evaluator counted down from three so that both readers would start simultaneously.

The first 200 syllables produced by the participants in each speech sample were analyzed using a Sony® VPC-AS laptop and Maxwell® HP200F headphones. Orthographic transcriptions were performed, and the stuttering episodes were marked and counted. Stuttering episodes were identified in accordance with the SSI-3 21, and the percentage of stuttered syllables in the samples was calculated. The average speech rate was also measured as the mean syllables spoken per minute.

### Statistical analyses

The collected data were analyzed using IBM SPSS Statistics 21.0. The data distribution was non-normal for all variables. Thus, the analyses were performed using non-parametric tests. In addition to the descriptive analysis, non-parametric inferential analyses were performed using Friedman's ANOVA test and Dunn's test for within group comparisons and the Mann-Whitney U test for between group comparisons. The significance level was set at 0.05.

## RESULTS

### Within group comparisons

Speech fluency measures were significantly different among speech tasks for Group 1 (Table 1). Choral reading produced a significantly lower percentage of stuttered syllables than the monologue task (*p*<0.001 according to Dunn's test) and oral reading (*p*<0.001 according to Dunn's test). Similarly, choral reading produced a higher number of syllables per minute than the monologue task (*p*<0.001 according to Dunn's test) and oral reading (*p*<0.001 according to Dunn's test). There were no significant differences between the monologue task and oral reading when considering both of the analyzed variables (*p*=1.00 for the percentage of stuttered syllables and for the number of syllables per minute; Dunn's test).

For Group 2, no significant differences were observed among the speech tasks for any of the investigated parameters (Table 2). Participants in Group 2 did not produce any stuttered syllables during the monologue task or choral reading. However, during the oral reading, two percent of one participant's syllables were stuttered.

### Between group comparison

The groups differed in all of the studied parameters. Group 1 had a higher percentage of stuttered syllables than Group 2 for all tasks. Group 1 produced a lower number of syllables per minute than Group 2 for all tasks. Between group comparisons revealed significant differences for all of the analyzed variables, including choral reading (Table 3).

## DISCUSSION

In this study, the performance of both fluent speakers and people who stutter was compared using three different speaking tasks: monologue, oral and choral reading. The study hypothesis (i.e., that choral reading would produce greater fluency for all speakers) was partially confirmed. The hypothesis was confirmed for individuals who stutter but was not conclusive for the fluent participants. As expected, the participants who stuttered had a lower stuttering frequency and faster speech rate during choral reading than during monologue and oral reading. Monologue and oral reading had similar frequencies of stuttering and speech rates, which were also expected.

Oral and choral readings are similar tasks (differing only in that there is an auditory stimulus during choral reading). For the participants who stutter, the percentage of stuttered syllables observed during the choral reading was approximately 70% lower than observed for oral reading. Additionally, speech rate was approximately 70% faster during choral reading. Therefore, according to the dual premotor system model 16,17, choral speech enhanced fluency by providing external cues for the timing of each syllable and compensated for deficient internal cues from the basal ganglia to the SMA. The observed fluency-enhancing effect of choral speech was consistent with previous studies 22,23,24.

Considering neuroimaging studies of the effect of choral speech on stuttered speech, a positron emission tomography (PET) study 25 of adults who stutter using both oral reading and monologue tasks observed positive correlations between the frequency of stuttering and the activation of the SMA, precentral gyrus, superior temporal gyrus and basal ganglia. The study also observed negative correlations between the frequency of stuttering and the activation of the lateral premotor cortex and cerebellum. Another study using functional magnetic resonance imaging (fMRI) compared stuttering speakers to non-stuttering controls during choral speech 26. The people who stuttered had a significantly greater increase in the activation of the superior temporal gyrus during choral speech. In contrast, the activation of the caudate, globus pallidus and putamen of the basal ganglia remained lower in stuttering speakers than in controls, even during choral speech.

According to the literature, the external stimuli delivered do not necessarily have to be delivered via the auditory system. Fluency enhancement can also occur when people who stutter are provided with visual feedback of targeted articulatory movements 27,28. However, not all external sensory stimuli can reduce stuttering. The stimulus needs to be perceived by the speaker as speech to reduce stuttering frequency. As long as the external signal is perceived as speech, the signal can engage the cerebellum and lateral premotor cortex shifting the motor control to the lateral system and inhibiting the speech disruptions 16.

Some limitations of the present study should be considered. Fluent participants were not able to improve upon the results observed during monologue and oral reading due to a ceiling effect masking a potential enhancement of fluency during choral speech and leading to inconclusive results for this group. The speech tasks used for this research were the standardized tasks used to assess the fluency of people who stutter at the Departamento de Fisioterapia, Fonoaudiologia e Terapia Ocupacional da Faculdade de Medicina da Universidade de São Paulo, São Paulo/SP, Brazil. These tasks may not have been ideally suited to measure fluency changes for fluent participants (therefore causing a ceiling effect). The ceiling effect still did not produce equivalent results for choral speech in participants who stutter and fluent participants. Nevertheless, the fluency performance results for the participants who stutter during choral speech were within normal limits 29.

Participants who stuttered had lower stuttering frequency and faster speech rate during choral reading than during the monologue task and oral reading. Thus, external sensory stimulation might contribute to the elucidation of the complex mechanisms of speech. The exact involvement of the basal ganglia and supplementary motor area during speech motor processing is not yet well-defined. Additional studies, especially neuroimaging studies, may provide some insight into the exact contribution of these structures to speech and, consequently, stuttering.

## AUTHOR CONTRIBUTIONS

Ritto AP was responsible for collecting, analyzing and tabulating the data and manuscript writing. Costa JB collaborated during the collection, analysis and tabulation of the data and during manuscript writing. Juste FS was responsible for supervising data collection and analysis and collaborated during manuscript writing. de Andrade CR was responsible for the study conception and design, she advised the other authors during the execution of the research and manuscript writing.

## ACKNOWLEDGMENTS

The authors thank the São Paulo Research Foundation (Fundação de Amparo à Pesquisa do Estado de São Paulo – FAPESP) for supporting this study, grants number 2011/10000-2 and 2014/05265-5.

## Figures and Tables

**Table 1:** Description of speech fluency characteristics in each task for Group 1.

Task	Percentage of stuttered syllables (%SS)				Number of syllables spoken per minute (syl/min)			
		Mean (SD)	Maximum	Minimum	*p-*value	Mean (SD)	Maximum	Minimum	*p-*value
Monologue	16.84 (7.82)	33	7.5	<0.001*	115.89 (42.09)	193.5	78.91	<0.001*
Oral Reading	12.91 (9.63)	33.5	3			131.04 (60.89)	272.72		44.44
Choral Reading	1.47 (2.45)	8	0			221.38 (63.21)	333.3		141.0

SD = Standard deviation;

**<?ENTCHAR ast?>:** = significant results (*p*<0.05) – Friedman's ANOVA test.

**Table 2:** Description of speech fluency characteristics in each task for Group 2.

Task	Percentage of stuttered syllables (%SS)				Number of syllables spoken per minute (syl/min)			
		Mean (SD)	Maximum	Minimum	*p*-value	Mean (SD)	Maximum	Minimum	*p*-value
Monologue	0.0 (0.0)	0	0	0.060 NS	235.78 (50.68)	285.71	146.34	0.058 NS
Oral Reading	0.21 (0.53)	2	0			332.36 (49.6)	400.0		240.0
Choral Reading	0.0 (0.0)	0	0			289.67 (14.69)	307.69		260.86

SD = Standard deviation; NS = not significant (*p*>0.05) – Friedman's ANOVA test.

**Table 3:** Between group comparisons of speech fluency characteristics.

		Percentage of stuttered syllables (%SS)				Number of syllables spoken per minute (syl/min)			
		Mean (SD)	U	Z	*p*-value	Mean (SD)	U	Z	*p*-value
Monologue	Group 1	16.84 (7.82)	0.0	-5.320	<0.001*	115.89 (42.09)	10.0	-4.639	<0.001*
		Group 2				0.0 (0.0)							235.78 (50.68)
Oral Reading	Group 1	12.91 (9.63)	0.0	-5.161	<0.001*	131.04 (60.89)	2.0	-4.913	<0.001*
		Group 2				0.21 (0.53)							332.36 (49.60)
Choral Reading	Group 1	1.47 (2.45)	68.0	-3.396	0.001*	221.38 (63.21)	51.0	-3.232	0.001*
		Group 2				0.0 (0.0)							289.67 (14.69)

Standard deviation;

**<?ENTCHAR ast?>:** = significant results (*p*<0.05) – Mann-Whitney U test.
